# Au@Cu Nanoarrays with Uniform Long-Range Ordered Structure: Synthesis and SERS Applications

**DOI:** 10.3390/mi9120678

**Published:** 2018-12-19

**Authors:** Pinhua Zhang, Haoming Sun, Wenhui Guan, Jinjin Liang, Xiaomeng Zhu, Junkai Zhang, Min Chen, Meng Cao, Wenbing Qian, Kefu Gao, Guangliang Cui

**Affiliations:** 1 School of Physics and Electrical Engineering, Linyi University, Linyi 276005, China; fuanliuzhong@163.com (W.G.); liangjinjin114@163.com (J.L.); lyuzhuxiaomeng@163.com (X.Z.); lyuchenmin@163.com (M.C.); fighting66cm@163.com (M.C.); qwenbsys@163.com (W.Q.); 2 School of Mechanical and Vehicle Engineering, Linyi University, Linyi 276005, China; lyusunhaoming@163.com (H.S.); gaokefu@lyu.edu.cn (K.G.); 3 Key Laboratory of Functional Materials Physics and Chemistry of the Ministry of Education, Jilin Normal University, Siping 136000, China; zjk@jlnu.edu.cn

**Keywords:** Cu nanoarrays, Au nanoparticles, surface-enhanced Raman scattering (SERS), surface decoration

## Abstract

The nanostructures with uniform long-range ordered structure are of crucial importance for performance standardization of high-quality surface-enhanced Raman scattering (SERS) spectra. In this paper, we described the fabrication and SERS properties of Au decorated Cu (Au@Cu) nanoarrays. The Cu nanoarrays with uniform long-range ordered structure were first synthesized by in-situ electrochemistry assembly on insulated substrate. The Cu nanoarrays can reach a size of centimeters with strictly periodic nano-microstructure, which is beneficial for the production and performance standardization of SERS substrates. Then Au nanoparticals were decorated on the Cu nanoarrays by galvanic reaction without any capping agent. The obtained Au@Cu nanoarrays exhibit excellent SERS activity for 4-Mercaptopyridine, and the sensitivity limit is as low as 10^−8^ M. Therefore, this facile route provides a useful platform for the fabrication of SERS substrates based on nano ordered arrays.

## 1. Introduction

Surface-enhanced Raman scattering (SERS) has attracted great attention in biological, chemical, and environmental fields because of its ultrasensitive fingerprint identification of chemicals [1,2,3,4,5]. The fast-accurate quantitative analysis of SERS is an important and necessary fundamental performance for practical applications, which is an unavoidable difficulty and challenge for further enhancement of practicality [6,7,8]. Two-dimensional nanomaterials with strictly ordered structures, i.e., ordered nanoarrays (NAs), in a large area (even up to the centimeter level) have obvious advantages for quantitative calculation due to their countable repeat unit of the structure. Hence, the design and preparation of SERS platform based on large ordered NAs become the key aspect of this problem.

Significant efforts have been focused on making ultra-sensitive SERS NAs including ZnO NAs, Au/TiO_2_ nanotube arrays and Au NAs [9,10,11,12,13]. Although the sensitivity of these NAs are generally very good, the non-strict order nanostructures make them difficult to achieve a highly level of technology standardization and quantitative analysis of SERS signals for the requirements of practical application. Thus, the main challenge is to fabricate SERS substrates based on large-size ordered NAs with countable repeat structure units. Meanwhile, noble metal (such as Au, Ag, Pt, Pd) decorated nanostructures have been recognized as promising functional materials with excellent activity for SERS application. Many corresponding nanostructures, including Au decorated TiO_2_ (Au@TiO_2_) nanocomposites, Au decorated Cu_2_O (Ag@Cu_2_O) nanostructures, Au decorated Cu (Ag@Cu) NAs, Au decorated Ag (Au@Ag) nanocubes and so on, have been proposed in the past few years [14,15,16,17,18]. Owing to the vast potential of noble metal and ordered NAs, the assembly of their nano-microstructure arrays into desired geometries is currently one of the most dynamic fields in nanotechnology [19]. Maybe the noble metal decorated large-size ordered NAs are ideal nanostructures for quantitative analysis of SERS.

In this work, we propose a facile route to prepare new Au@Cu NAs with relatively strictly ordered nanostructure in the order of sub-centimeters. The Cu NAs were first synthesized by template-free in-situ electrochemistry assembly on the substrate, then Au nanoparticles (NPs) were decorated on the Cu NAs by galvanic reaction without any capping agent. The Au@Cu NAs can reach a size of centimeters with strictly periodic nano-microstructure, which is benefit for the production and performance standardization of SERS substrates. The pollution-free surface of the Au@Cu NAs could also provide a powerful field-enhancement for SERS performance. This strategy is suitable for the preparation of noble metal decorated large-size ordered NAs, which will pave a way to achieve nanostructures with desired geometries for applications in SERS, therapy, environmental protection and catalysis systems.

## 2. Materials and Methods 

### 2.1. Materials

Copper sulfate (CuSO_4_), sodium chloraurate (NaAuCl_4_), 4-Mercaptopyridine (4-Mpy), l-cysteine (L-Cys), methyl orange (MO) and rhodamine 6G (R6G) were purchased from Aladdin (Aladdin, Shanghai, China). All the reagents were used as received without further purication. The solutions were prepared with deionized water. Two copper foils (30 μm thick, 2 mm wide) were used as electrodes.

### 2.2. Synthesis of Au Decorated Cu Ordered Nanoarrays (Au@Cu NAs)

Cu NAs were prepared by in-situ electrochemistry assembly method. In a typical electrochemistry process, 0.499 g CuSO_4_ was dissolved in 50 mL deionized water, and 25 μL CuSO_4_ solution was dropped on the Si substrate between two parallel copper electrodes (separated by a distance of 6 mm). Then the electrolyte was frozen and an ultra-thin liquid layer of concentrated electrolyte formed between the ice layer and the Si substrate. After that, deposition was carried out by applying a 0.8 Hz semi-sine wave potential with an amplitude varied from 0.5 V to 1.1 V. When the growth process finished, the Cu NAs formed on the surface of substrate were taken out, and cleaned by deionized water. At last, the obtained Cu NAs were immersed into 10^−4^ M NaAuCl_4_ solution for specific time (1–5 min), then taken out and washed three times with deionized water.

### 2.3. Surface-Enhanced Raman Scattering (SERS) Measurements

For SERS experiments, 4-Mpy was used as the probe molecule. 4-Mpy solution was dropped onto the Au@Cu NAs and dried naturally in air at room temperature. The SERS spectra of the Au@Cu NAs samples were collected by a confocal Renishaw Raman microscope system with a 785 nm excitation laser. The laser power used was 1.5 mW which was focused on to the sample by a 50× objective lens, and the integral time was 10 s.

### 2.4. Characterization

The morphology and structure of Au@Cu NAs were examined by a scanning electron microscope (SEM, EVO 18, ZEISS, Oberkochen, Germany) and a transmission electron microscope (TEM, JEM-2200FS, JEOL, Tokyo, Japan). The elemental composition and chemical state of the Au@Cu NAs were determined by X-ray photoelectron spectroscopy (XPS) using a Thermo Scientic ESACLAB 250Xi system.

## 3. Results and Discussion

**Figure 1:**
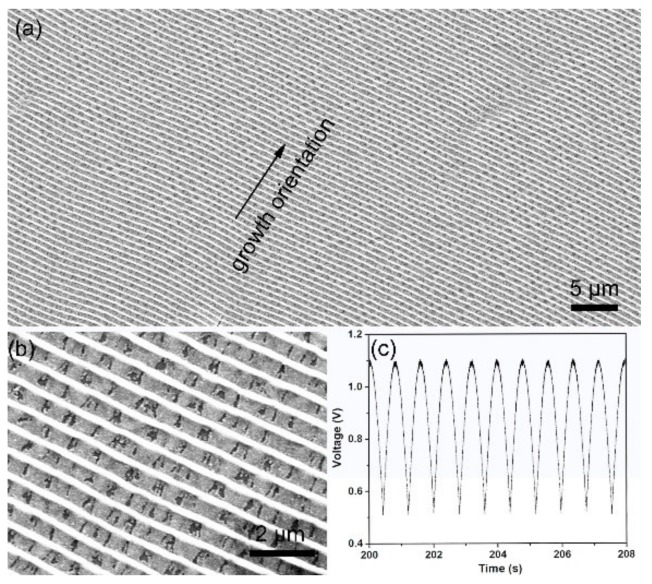
The typical (**a**) low- and (**b**) high-magnification SEM images of Au decorated Cu (Au@Cu) nanoarrays; (**c**) semi-sine wave voltage applied across the electrodes in the electrochemical deposition process.

**Figure 2:**
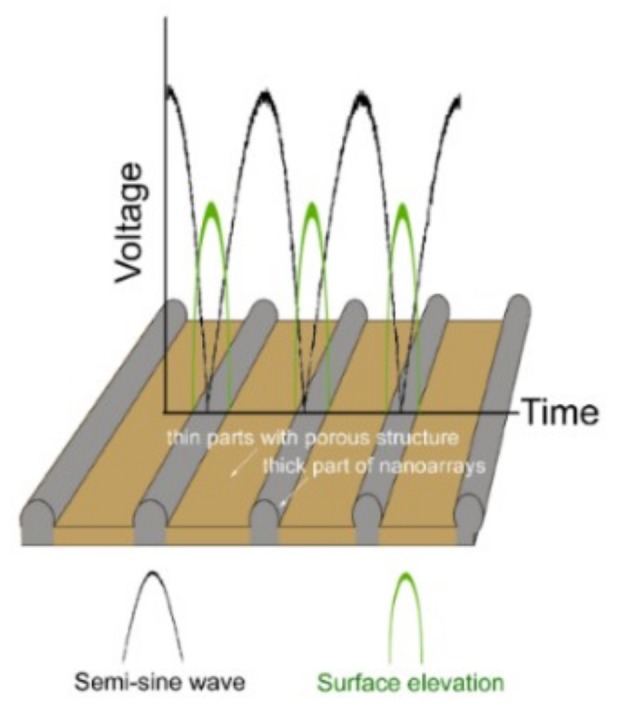
Schematic diagrams showing the periodic deposition process. The growth process at the lower and upper part of the semi-sine wave potential are corresponding to the deposition of thick and thin parts.

The SEM images of the obtained Au@Cu NAs are shown in Figure 1. Highly ordered Cu NAs were achieved after an in-situ electrochemistry assemble process without any template. Figure 1a shows a top view of the Au@Cu NAs over a large area, the wavy pattern exhibits uniform cycle length and good long-range order. The length of Au@Cu NAs can reach one centimeter, leading to great advantages in practical application, such as the preparation and performance standardization of nano-microstructure detection elements. As we can see in Figure 1b, the pattern is porous film with a strictly nano-ordered structure. Figure 1c shows the semi-sine wave voltage applied in the electrochemistry assembly process, the offset and amplitude are 0.5 V and 0.6 V, the frequency is 0.8 Hz. The periodicity of the deposition voltage takes primary responsibility for the wavy pattern. As shown in Figure 2, the growth velocity is closely related to the concentration of electrolyte and the applied potential, and the cyclic growth pattern is caused by the variation of ion concentration near the growth front lagging behind the variation of electrode potential. In a typical deposition process, when the potential was low, Cu^2+^ could migrate to the growth front in time as the deposition process was relatively slow. Therefore, the formed nanocrystals were easy to accumulate. While the deposition process was relatively fast with high potential, Cu^2+^ could not migrate to the growth front rapidly enough to keep up with the rate of deposition, thus the nanocrystals formed with mild accumulation. This is also the reason for the formation of the porous structure. Finally, this periodic deposition process led to the periodic variation in surface elevation of Cu nanoarrays [20,21,22,23].

**Figure 3:**
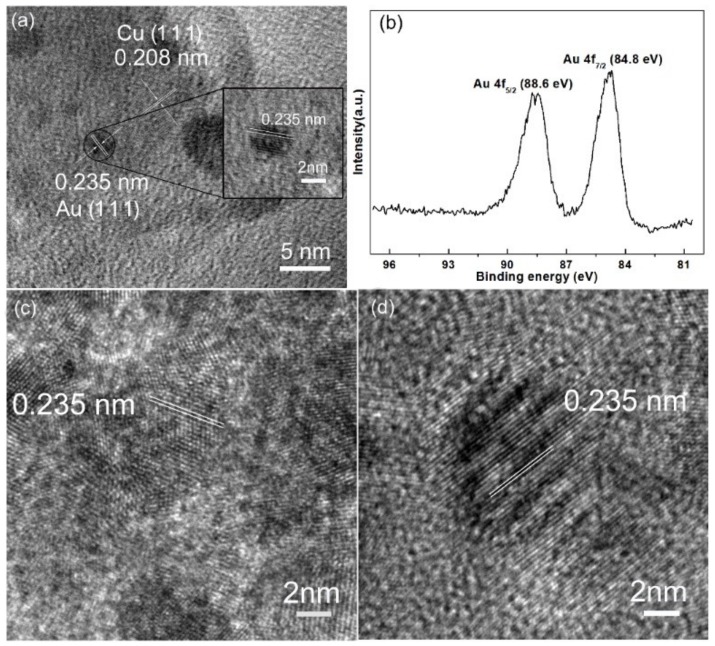
(**a**) High-resolution transmission electron microscopy (HRTEM) and (**b**) High-resolution XPS spectrum of Au 4f of Au@Cu nanoarrays with galvanic reaction time of 3 min. (**c**,**d**) are HRTEM images of Au@Cu nanoarrays with galvanic reaction time of 1 min and 5 min.

The Cu NAs were immersed into 10^−4^ M NaAuCl_4_ solution for specific time, then Au NPs would form on the surface of Cu NAs through galvanic reaction process. The SEM results reveal that the pattern of Au@Cu NAs has not been changed after galvanic reaction process, however, we can prove the formation of Au NPs by HRTEM and XPS (Figure 3). The HRTEM image of the Au@Cu NAs is shown in Figure 3a. As we can see that the size of Au NPs is about 3 nm, while the spacing of the fringes is measured to be 0.235 nm corresponding to the (111) plane of Au NPs. Meanwhile the spacing of the background fringe is measured to be 0.208 nm, corresponding to the (111) plane of Cu. XPS was used to further prove the formation of Au NPs, as shown in Figure 2b. The Au XPS spectrum shows peaks at binding energies of 88.6 and 84.8 eV, which are attributed to metallic Au 4f_5/2_ and Au 4f_7/2_, respectively [24]. The above results are powerful proofs of the existence of the metallic Au NPs on the surface of Cu NAs. The HRTEM images of the Au@Cu NAs with galvanic reaction time of 1 min and 5 min are shown in Figure 3c,d. As shown in Figure 3c, it is obvious that the galvanic reaction time of 1 min is not enough for the deposition of Au NPs. The Au NPs are too small with fuzzy lattice. When prolonging the immersion time to 3 min, there are many Au NPs immobilized on the arrays, and the Au NPs are small in size with apparent lattice, as shown in Figure 3a. The Au NPs are larger and sparser as the galvanic reaction time further increased to 5 min, as shown in Figure 3d. Therefore, Au@Cu nanoarrays with galvanic reaction time of 3 min could supply more hot spots for SERS.

**Figure 4:**
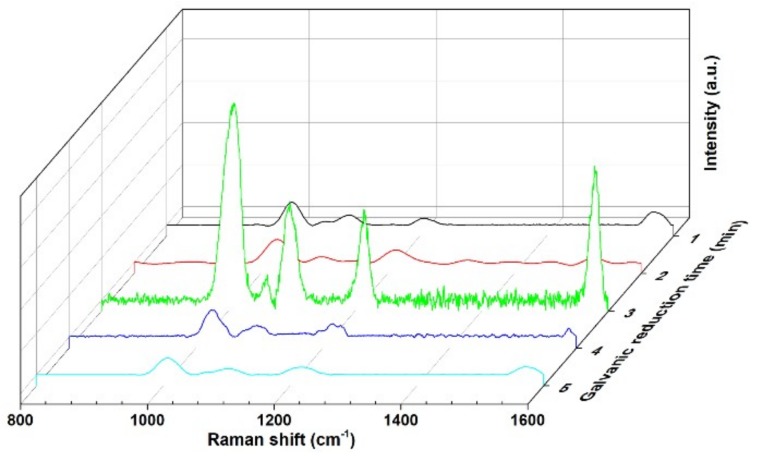
Surface-enhanced Raman scattering (SERS) spectra of 4-Mercaptopyridine (4-Mpy) absorbed on Au@Cu nanoarrays with galvanic reaction time ranging from 1 min to 5 min.

4-Mpy has bifunctional groups of sulfhydryl (thiol) and pyridyl, which provide three possible ways for the interaction between 4-MPy and the metal surface, namely via the lone pair electrons of the S or N atom or via the π electrons. The unpaired electron of sulfur atoms in 4-Mpy is known to exhibit strong electronic affinity to metal surface (especially Au, Ag, and so on) and presents strong and characteristic SERS signals, so the SERS activity of the Au@Cu NAs was evaluated by employing 4-Mpy as the probe molecule [25,26]. As shown in Figure 4, the SERS performances of the Au@Cu NAs with decoration time ranging from 1 min to 5 min were measured, and the strongest Raman signal was detected when the Cu NAs were immersed in NaAuCl_4_ solution for 3 min. Moreover, we found that SERS activity of the Au@Cu NAs was highly dependent on the time of the galvanic reaction. The SERS activity of the Au@Cu NAs was enhanced with an increase in galvanic reaction time from 1 min to 3 min. However, with further increasing the time from 4 min to 5 min, the SERS activity was instead diminished. The results are well agreed with the TEM conclusion in Figure 3.

**Figure 5:**
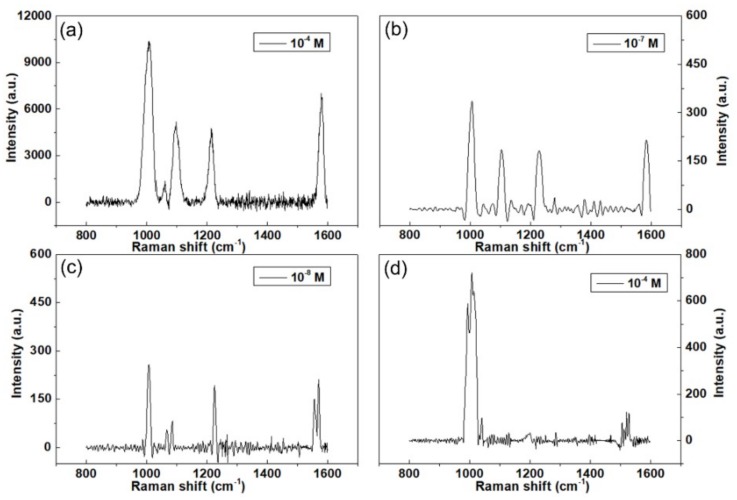
(**a**–**c**) Concentration-dependent (10^−4^ M, 10^−7^ M and 10^−8^ M) SERS spectra of 4-Mpy adsorbed on the Au@Cu NAs with the galvanic reaction time of 3 min. (**d**) SERS spectrum of 10^−4^ M 4-Mpy absorbed on Cu NAs.

Figure 5a–c show SERS spectra of 4-Mpy at different concentrations (10^−4^, 10^−7^ and 10^−8^ M) adsorbed on Au@Cu NAs with the galvanic reaction time of 3 min. We can see from Figure 5a–c that the intensity of the SERS signal gradually decreased with the decrease of the concentration of 4-Mpy. Even at 10^−8^ M, the SERS spectrum of 4-Mpy shows obvious peaks, which means that the synthesized Au@Cu NAs were able to detect 4-Mpy at a concentration of 10^−8^ M. Moreover, the SERS peaks of 10^−4^ M 4-Mpy absorbed on Cu NAs could also be identified, as shown in Figure 5d, but the intensity is much lower than that of the Au@Cu NAs. This enhanced sensitivity of 4-Mpy detection can be attributed to several factors. First, due to the wavy pattern possessing high specific surface area, the Au@Cu NAs provide adequate adsorption surfaces for 4-Mpy molecules; second, the Au NPs decorated on Cu NAs through galvanic reaction without any capping agent, so the surface of the Au@Cu NAs is clean, which is also contributed to the excellent SERS performance. Besides, SERS activity of Au is much better than Cu [27], especially when the Au NPs is small in size [28]. Therefore, by coating a thin layer of Au NPs on top of the Cu substrates, the SERS sensitivity is enhanced.

**Figure 6:**
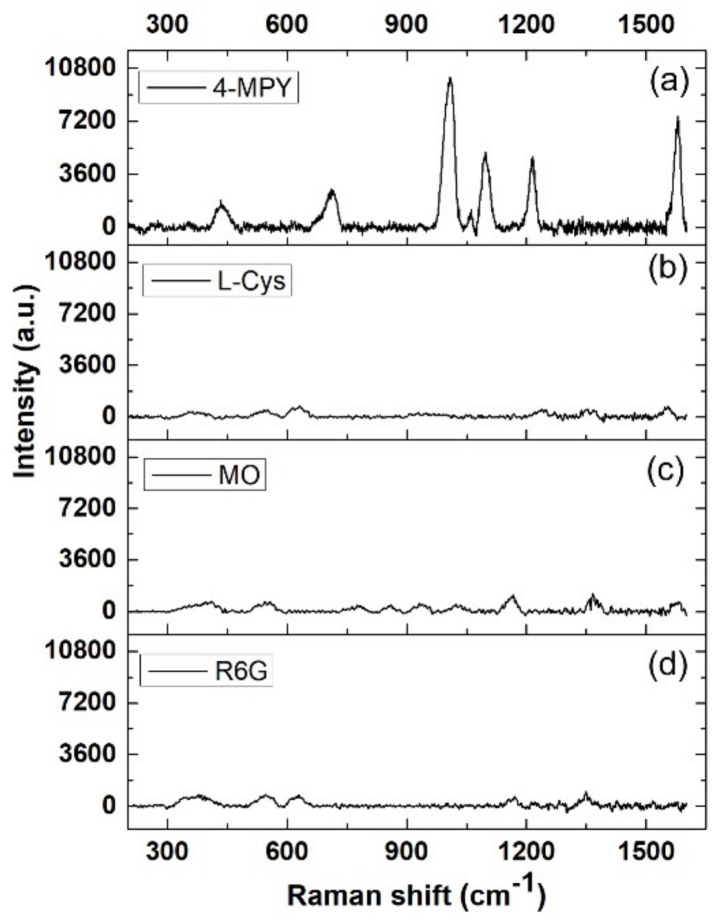
Selectivity of the SERS substrate with galvanic reaction time of 3 min. The concentrations of (**a**) 4-Mpy, (**b**) l-cysteine (L-Cys), (**c**) methyl orange (MO) and (**d**) rhodamine 6G (R6G) are 10^−4^ M.

To evaluate the selectivity of our SERS substrate, three analytes (L-Cys, MO and R6G) were immobilized on the SERS substrates at the same concentration of 10^−4^ M. As shown in Figure 6, very weak Raman signals were detected for L-Cys, MO and R6G according to the same quantitative method. The 4-Mpy molecule has a sulfhydryl group (S-H), which could be combined at the surface of gold through Au-S chemical bonds and presents strong SERS signals [25]. While other three molecules have sulfur and/or nitrogen containing functional groups, however the SERS signals are not significant. In conclusion, Au NPs on the surface of Cu NAs provide potential binding sites for sulfhydryl functional groups via a specific Au–S bond and consequently exhibit high selectivity for 4-Mpy. These results demonstrate that our SERS substrate has high selectivity against these analytes, indicating that the detection of 4-Mpy is unaffected in spite of the presence of these interferons.

## 4. Conclusions

In summary, we have demonstrated the potential of using a highly ordered Au@Cu NAs as SERS platform for detection of 4-Mpy. This SERS platform with long-range ordered nano-microstructure on the centimeter scale was synthesized by a template-free method. The obtained SERS substrate has the advantages of easy fabrication, high specific surface area and clean surface. The Au@Cu NAs with galvanic reaction time of 3 min exhibit the greatest SERS activity to 4-Mpy, and it can be applied to detect 4-Mpy with a detection limit of 10^−8^ M. Besides, the Au@Cu NAs show high selectivity and specificity for 4-Mpy detection. Therefore, it is possible for applications in environmental protection and analysis of unique one of a kind materials.
